# Lenalidomide Induces Lipid Raft Assembly to Enhance Erythropoietin Receptor Signaling in Myelodysplastic Syndrome Progenitors

**DOI:** 10.1371/journal.pone.0114249

**Published:** 2014-12-03

**Authors:** Kathy L. McGraw, Ashley A. Basiorka, Joseph O. Johnson, Justine Clark, Gisela Caceres, Eric Padron, Ruth Heaton, Yukiyasu Ozawa, Sheng Wei, Lubomir Sokol, Alan F. List

**Affiliations:** 1 Department of Malignant Hematology, H. Lee Moffitt Cancer Center, 12902 Magnolia Drive, Tampa, FL 33612, United States of America; 2 Department of Malignant Hematology, H. Lee Moffitt Cancer Center-Cancer Biology Ph.D. Program, University of South Florida, 12902 Magnolia Drive, Tampa, FL, 33612, United States of America; 3 Analytic Microscopy Core Facility, H. Lee Moffitt Cancer Center, 12902 Magnolia Drive, Tampa, FL, 33612, United States of America; 4 Morsani Molecular Diagnostic Laboratory, H. Lee Moffitt Cancer Center, 10902 N. McKinley Drive, Tampa, FL, 33612, United States of America; 5 Department of Pathology, University of Arizona, 1501 N Campbell Ave, Tucson, AZ, 85724, United States of America; 6 Department of Hematology, Japanese Red Cross Nagoya First Hospital, 3-35 Michishita-cho, Nakamura-ku, Aichi, 453-8511, Japan; 7 Department of Immunology, H. Lee Moffitt Cancer Center, 12902 Magnolia Drive Tampa, FL, 33612, United States of America; Cincinnati Children's Hospital Medical Center, United States of America

## Abstract

Anemia remains the principal management challenge for patients with lower risk Myelodysplastic Syndromes (MDS). Despite appropriate cytokine production and cellular receptor display, erythropoietin receptor (EpoR) signaling is impaired. We reported that EpoR signaling is dependent upon receptor localization within lipid raft microdomains, and that disruption of raft integrity abolishes signaling capacity. Here, we show that MDS erythroid progenitors display markedly diminished raft assembly and smaller raft aggregates compared to normal controls (p = 0.005, raft number; p = 0.023, raft size). Because lenalidomide triggers raft coalescence in T-lymphocytes promoting immune synapse formation, we assessed effects of lenalidomide on raft assembly in MDS erythroid precursors and UT7 cells. Lenalidomide treatment rapidly induced lipid raft formation accompanied by EpoR recruitment into raft fractions together with STAT5, JAK2, and Lyn kinase. The JAK2 phosphatase, CD45, a key negative regulator of EpoR signaling, was displaced from raft fractions. Lenalidomide treatment prior to Epo stimulation enhanced both JAK2 and STAT5 phosphorylation in UT7 and primary MDS erythroid progenitors, accompanied by increased STAT5 DNA binding in UT7 cells, and increased erythroid colony forming capacity in both UT7 and primary cells. Raft induction was associated with F-actin polymerization, which was blocked by Rho kinase inhibition. These data indicate that deficient raft integrity impairs EpoR signaling, and provides a novel strategy to enhance EpoR signal fidelity in non-del(5q) MDS.

## Introduction

The myelodysplastic syndromes (MDS) include a spectrum of hematopoietic stem cell malignancies that share features of cytological dysplasia and ineffective hematopoiesis. Bone marrow progenitors from patients with MDS display diminished STAT5 activation and transcriptional response to erythropoietin (Epo) stimulation compared to age matched controls despite normal Epo receptor (EpoR) membrane density [1], [2]. The precise mechanisms underlying the impairment in cytokine signaling remain unclear. Fuhler et al. previously reported that granulocyte-macrophage colony stimulating factor (GM-CSF) priming was significantly reduced in neutrophils from MDS patients, owing in part to deficient membrane lipid raft formation [3]. Lipid rafts are specialized membrane microdomains that consolidate signaling intermediates to produce focused signaling platforms. We recently reported that EpoR signaling is dependent upon receptor residence within membrane lipid rafts and that raft disruption abolished Epo signaling [4]. Erythropoietin induced the formation and aggregation of lipid rafts, as well as the recruitment of key signaling intermediates such as EpoR, JAK2, STAT5, and Lyn kinase. Furthermore, receptor engagement by erythropoietin triggered translocation of the signal-attenuating transmembrane tyrosine phosphatase, CD45, to non-raft domains, thereby potentiating signal capacity [4]. Disruption of rafts by membrane cholesterol depletion inhibited Epo-induced STAT5 activation in both erythroid cell lines and primary bone marrow erythroid progenitors, confirming the critical role of raft integrity in cellular Epo response [4]. Furthermore, inhibition of Rho and Rac GTPases, important regulators of the actin cytoskeleton, blocked recruitment of EpoR into the raft fractions, indicating a key role for these proteins in the coordination of EpoR membrane domain localization [4].

GTPases are activated by immunomodulatory agents (IMiDs), which in turn trigger assembly of the immune synapse in T- and NK-cells [5]–[9]. The second generation IMiD, lenalidomide, improves erythropoiesis and promotes red blood cell transfusion independence in approximately two thirds of del(5q) MDS patients by directly suppressing the malignant clone. However, in lower risk non-del(5q) MDS approximately 25% of patients achieve transfusion independence by a mechanism in which lenalidomide promotes effective erythropoiesis in the MDS clone [10]–[12]. Ebert et al. showed that responding non-del(5q) patients under-expressed a set of erythroid differentiation genes whose expression was restored after lenalidomide exposure, indicating that lenalidomide may modify inherent limitations in EpoR signaling and transcriptional response [2]. To elucidate mechanisms underlying diminished EpoR signal capacity in MDS and discern strategies to improve signal fidelity, we investigated membrane lipid raft integrity in bone marrow erythroid progenitors from patients with lower risk MDS. Our findings show that MDS erythroid progenitors are deficient in membrane lipid rafts, and that treatment with lenalidomide improves raft assembly to enhance EpoR signaling and colony forming capacity.

## Results

### MDS erythroid precursors are deficient in lipid rafts

**Figure 1:**
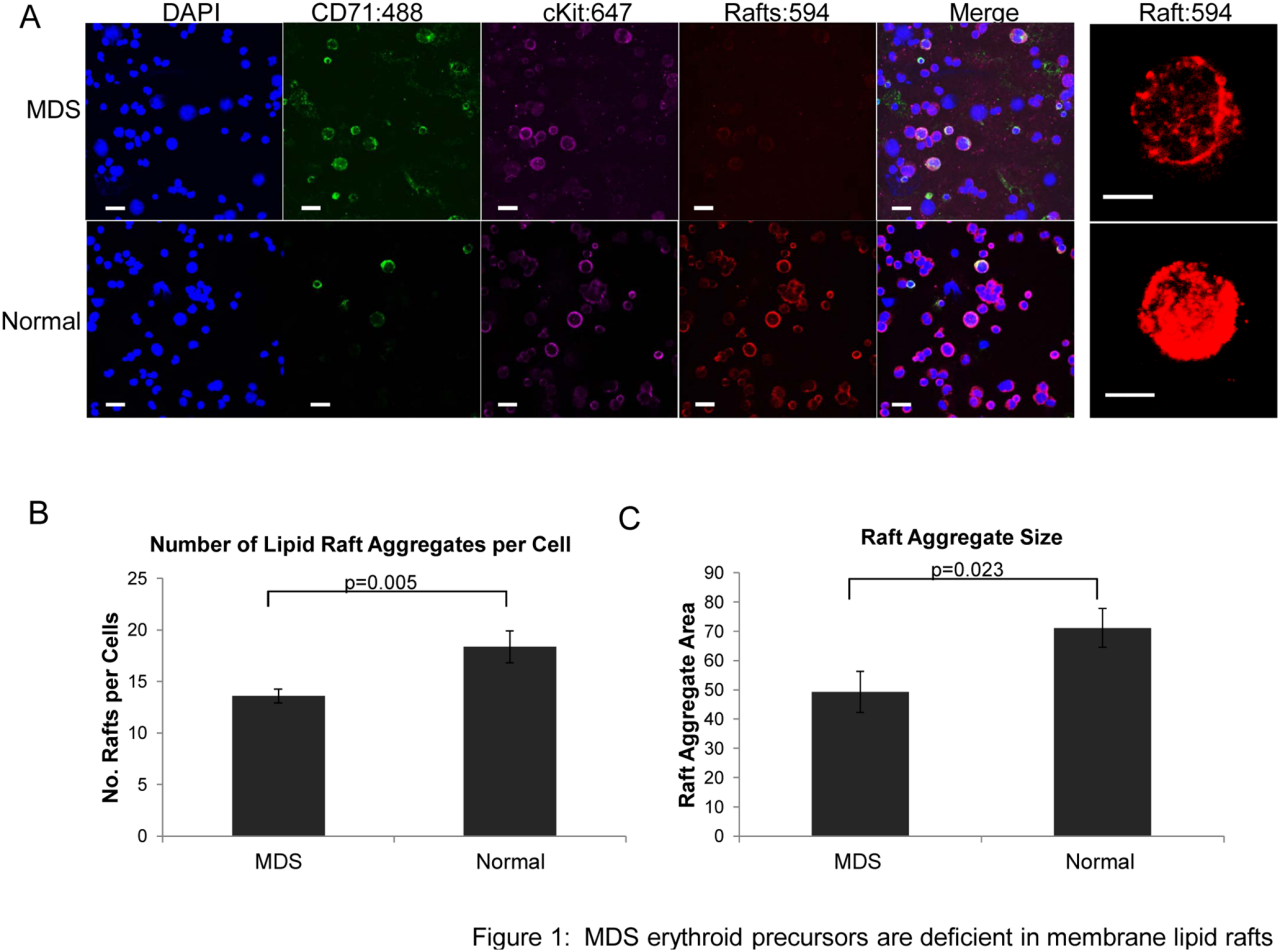
MDS erythroid precursors are deficient in membrane lipid rafts. (A) Representative micrographs of primary erythroid precursors from an MDS patient (top) compared to normal donor (bottom) erythroid progenitors identified by confocal immunofluorescence microscopy. Primitive erythroids express CD71+ (green) and cKit+ (purple); lipid rafts display red fluorescence, DAPI (blue), merged image is shown in the last panel. Bars represent 25 µm. Enlarged images illustrate lipid rafts at 630x with 6-zoom magnification. Bars represent 5 µm. (B) Statistically significant decrease in the number of rafts per cell in MDS erythroid precursors compared to normal donor erythroid cells, graph represents mean ± SE. (C) Statistically significant decrease in the size of raft aggregates in primary erythroids compared to normal erythroids. Mean ± SE.

We first sought to determine whether integrity of membrane lipid raft assembly and/or EpoR partitioning within rafts limits Epo responsiveness in MDS erythroid progenitors. Primary bone marrow mononuclear cells (BM-MNC) were isolated from 11 IPSS low/intermediate-1 risk, non-del(5q) MDS patients (clinical characteristics of the patients are summarized in Table 1, gene mutation analysis was not performed) following written on IRB approved protocols and from 3 normal donors. Cytospin preparations were stained with CD71 and cKit antibodies, and CT-B:594 conjugate that binds GM-1, a raft constituent ganglioside whose fractionation and membrane localization identifies lipid rafts (Figure 1A). Erythroid progenitors were identified as dual CD71+ and cKit+ cells. The number of raft clusters and size of raft aggregates were determined by confocal microscopy using automated software. Erythroid precursors from all MDS patients (erythroid cell numbers ranged from 8–119 cells per patient) and from all normal donors (erythroid cell numbers ranged from 55–135 per donor) were pooled for analysis [total cell number: MDS, n = 617; normal donors, n = 333]. Mean raft number per cell was significantly lower in MDS erythroid cells compared to normal donor erythroids [13.60±0.67 (mean ± SE) and 18.37±1.56, respectively, p = 0.005] (Figure 1B). Furthermore, the average area of the raft aggregates was significantly reduced in MDS erythroid precursors compared to normal erythroids (49.31±6.98 vs. 71.17±6.63, respectively, p = 0.023) (Figure 1C). These findings of markedly reduced membrane rafts as well as raft aggregate size in MDS erythroid precursors may limit EpoR signal capacity and contribute to the impairment in EpoR signaling.

**Table 1:** Clinical characteristics of MDS patients.

MDS Sample Number	Response tolenalidomide	Cytogenetics	IPSS	Diagnosis	Age
1	NE	NA	NA	NA	NA
2	Yes	45, X, –Y [20]	Int-1	RARS	89
3	Yes	46, XY [20]	Low	RARS	78
4	No	46, XY, INV(11q21; q23) [20]	Int-1	RCMD	71
5	Yes	46, XY [20]	Low	RCMD	79
6	NE	46, XX [20]	Low	RARS	73
7	No	NA	NA	NA	80
8	Yes	46, XY [20]	Low	RARS	66
9	No	46, XY [3];45, X, –Y [17]	Low	RARS	81
10	Yes	46, XY [20]	Int-1	RA	76
11	NE	46, XY [20]	Low	RARS	71

IPSS: International prognostic scoring system, RA: refractory anemia, RARS: refractory anemia with ringed sideroblasts, RCMD: Refractory cytopenia with multilineage dysplasia, Int-1: Intermediate-1, NE: Non-evaluable, NA: Not available.

### Lenalidomide induces raft aggregation in UT7 cells and MDS erythroid progenitors

**Figure 2:**
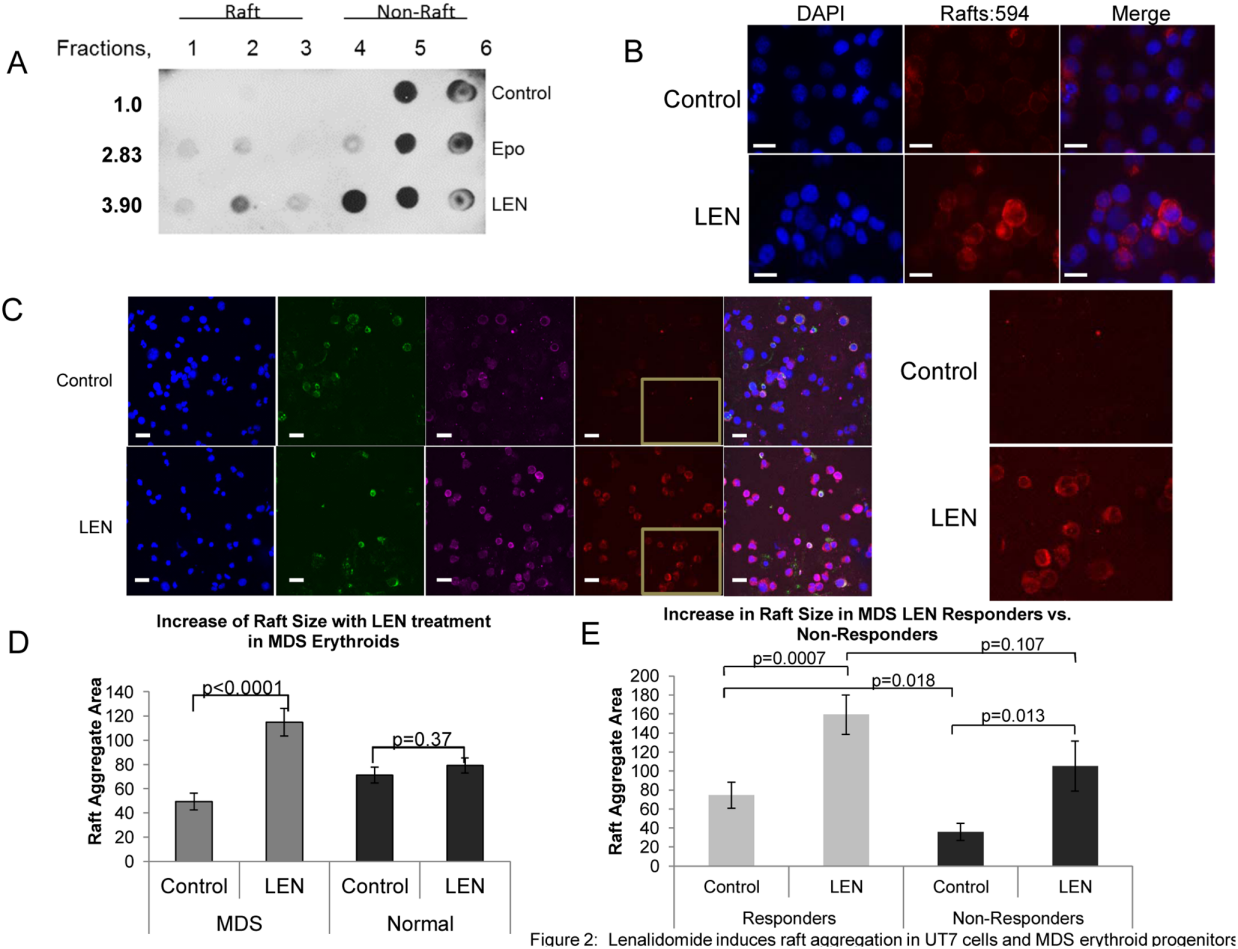
Lenalidomide induces raft aggregation in UT7 cells and MDS erythroid progenitors. (A) Dot blot detection of GM-1 membrane fractionation in UT7 cells treated with 1 U/ml Epo or 1 µM LEN for 1 hr. Rafts are located predominantly in fraction 2 (all raft fractions 1–3) whereas non-raft fractions are represented by fractions 4–6. Numbers represent densitometry analysis of combined raft fractions 1–3. (B) Immunofluorescence of UT7 cells, DAPI (blue), rafts (red), and merged image showing marked accumulation of rafts after lenalidomide treatment. Bars represent 20 µm. (C) Representative micrograph of MDS primary erythroid precursors identified by CD71+ (green) and cKit+ (purple) expression, untreated (top) and lenalidomide treated (bottom) showing increased raft aggregation following drug treatment. Bars represent 25 µm. Enlarged image from inset shown. (D) There is a statistically significant increase in the size of raft aggregates of MDS erythroid cells that is not observed in normal erythroid cells. (E) Comparison of lipid raft size in lenalidomide responding MDS patient erythroid precursors compared to non-responders.

Previously we showed that treatment of the erythroid progenitor cell line, UT7 with recombinant human erythropoietin (rhEpo) stimulated raft assembly within minutes of growth factor exposure [4]. Given the findings that lenalidomide induces immune synapse formation in T-lymphocytes, we investigated whether lenalidomide would promote lipid raft assembly in MDS erythroid precursors and UT7 cells. To evaluate this, we first treated UT7 cells with 1 µM lenalidomide for 1 h, the previously determined optimal pretreatment incubation period, and isolated lipid rafts by ultracentrifugation. Cellular fractions were dot blotted using cholera toxin B (CT-B):HRP conjugate for GM-1 (Figure 2A). [13]–[15] Treatment with lenalidomide increased GM-1 positive membrane fractions (fractions 1–3), indicating that lenalidomide promoted membrane lipid raft assembly. Importantly, the increase in rafts exceeded that observed following rhEpo stimulation (Figure 2A). Interestingly, combined treatment with rhEpo and LEN was not additive, and in fact resulted in a lower magnitude of induction in raft assembly compared to lenalidomide alone, suggesting that rhEPO and lenalidomide augment membrane raft assembly by distinct mechanisms. To confirm that the increase in raft fractions after lenalidomide exposure reflected induction of raft formation, we assessed the number and size of lipid rafts by GM-1 immunofluorescence (Figure 2B). Immunofluorescence data confirmed the induction of membrane rafts by lenalidomide. Collectively, these results demonstrate that LEN is able to stimulate raft formation and aggregation independent of cytokine or receptor stimulation.

To determine if lenalidomide also induces rafts in primary erythroid progenitors, BM-MNC from MDS patients (n = 11) and normal donors (n = 3) were treated with 1 uM lenalidomide for 1 h prior to raft and surface marker staining (Figure 2C). Analysis of CD71+/cKit+ erythroid cells from MDS patients demonstrated an increase in the total raft number per cell that did not reach statistical significance (13.60±0.67 vs. 15.30±1.05, p = 0.18). However, the size of the membrane raft aggregates significantly increased after LEN treatment (mean area per raft 49.31±6.97 vs. 114.77±11.36, respectively, p<0.0001) (Figure 2D). These results indicate that lenalidomide induces aggregation of smaller raft clusters into larger membrane signaling platforms that may potentiate EpoR signaling. In normal erythroid cells, lenalidomide did not significantly alter the size of raft aggregates (p = 0.37; Figure 2D), suggesting that the potentiating effect on raft assembly in MDS arises from actions on aberrant mechanisms limiting raft formation in raft deficient MDS erythroid cells. Of the 11 MDS patients analyzed, 8 received treatment with lenalidomide, including 5 responders and 3 non-responders. Membrane raft size was significantly larger in the erythroid progenitors of responding patients compared to non-responders (n = 242 and n = 194, p = 0.018) (Figure 2E). Furthermore, lenalidomide increased raft size in responders [mean raft area 74.52 for untreated (n = 242) and 159.44 for lenalidomide treated (n = 290) erythroid progenitors] (p = 0.0007) and non-responders [mean raft area 35.85 for untreated (n = 194) compared to 105.25 for lenalidomide treated (n = 140) erythroid progenitors] (p = 0.013) with greater raft induction in responding patient progenitors, (p = 0.11) (Figure 2E).

### Lenalidomide recruits EpoR signal effectors into raft fractions

**Figure 3:**
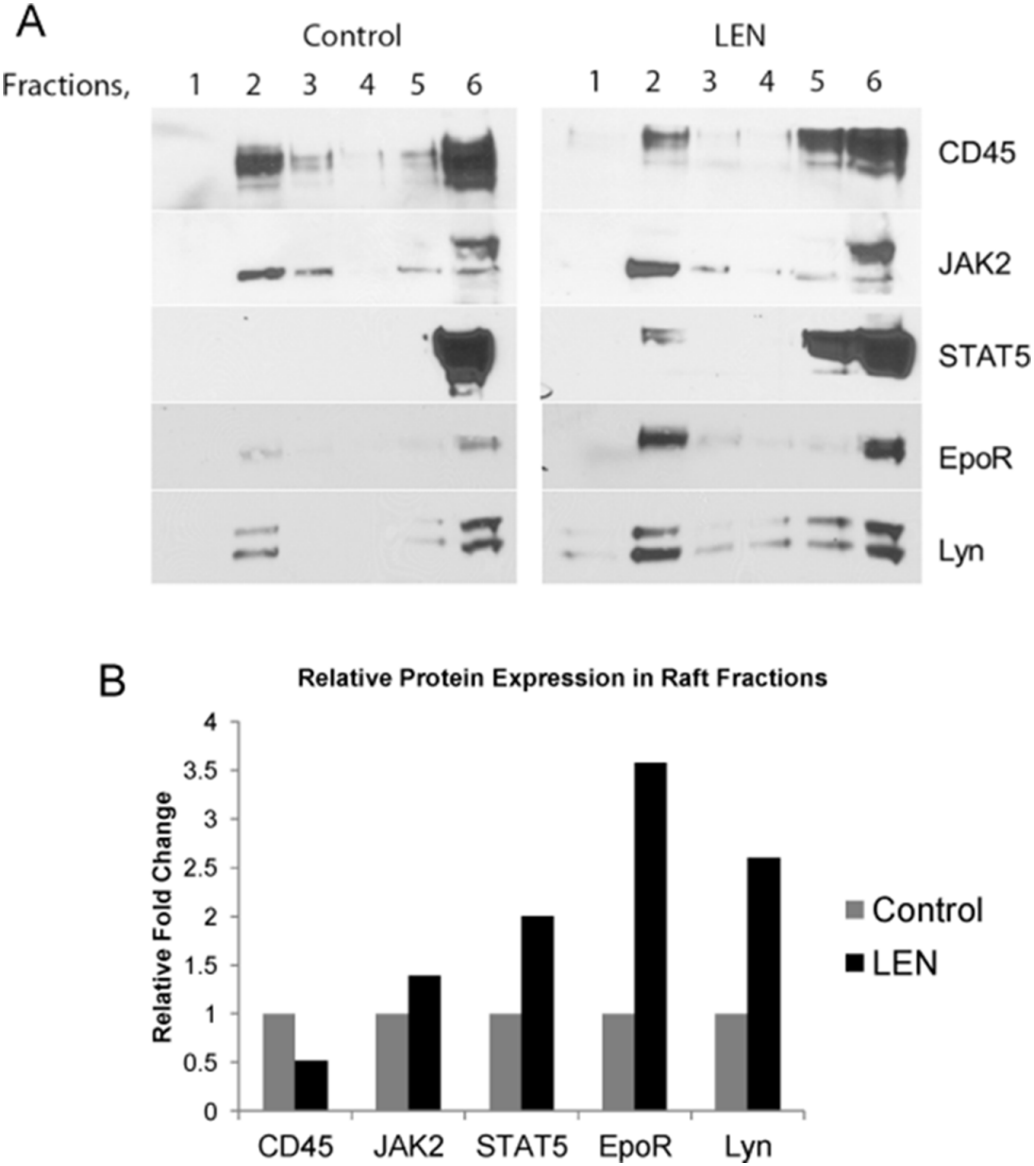
Lenalidomide induces recruitment of EpoR and signaling effectors into lipid rafts. (A) Western blot of cell fractions showing lipid rafts primarily in fraction 2 and non-raft fractions in 5 and 6. Lenalidomide induces recruitment of EpoR, JAK2, and STAT5 into raft fractions while displacing CD45. Lyn kinase serves as an additional marker for lipid raft fractionation, and is also increased in raft fractions after lenalidomide treatment. (B) Relative raft protein expression by densitometry analysis.

Our prior studies of rhEpo showed that in addition to inducing raft formation and aggregation, rhEpo induced the recruitment of EpoR and its signaling intermediates JAK2, STAT5, and Lyn kinase into raft fractions. Treatment with rhEpo also partitioned the negative regulator and transmembrane protein, tyrosine phosphatase, CD45, out of the raft fractions, thereby potentiating fidelity of the EpoR signal [4]. To determine whether lenalidomide treatment effected raft constituents, we treated UT7 cells with lenalidomide and isolated both the raft (fraction 1–3) and non-raft fractions (fractions 4–6) after ultracentrifugation (fractions were confirmed by dot blot, data not shown). These fractions were then probed by western blot analysis (Figure 3A and B). Lenalidomide induced the recruitment of EpoR into lipid raft fractions after 1 hr of drug exposure. Furthermore, JAK2, STAT5, and Lyn kinase (whose fractionation also acts as a marker for lipid rafts) showed increased partitioning with GM-1 after lenalidomide treatment, indicating recruitment of signaling effectors into discrete signaling platforms. Lastly, we found that treatment with lenalidomide also redistributed CD45 to non-raft fractions, which may further enhance signal fidelity (Figure 3A and B).

### Lenalidomide enhances EpoR signaling in erythroid progenitors

**Figure 4:**
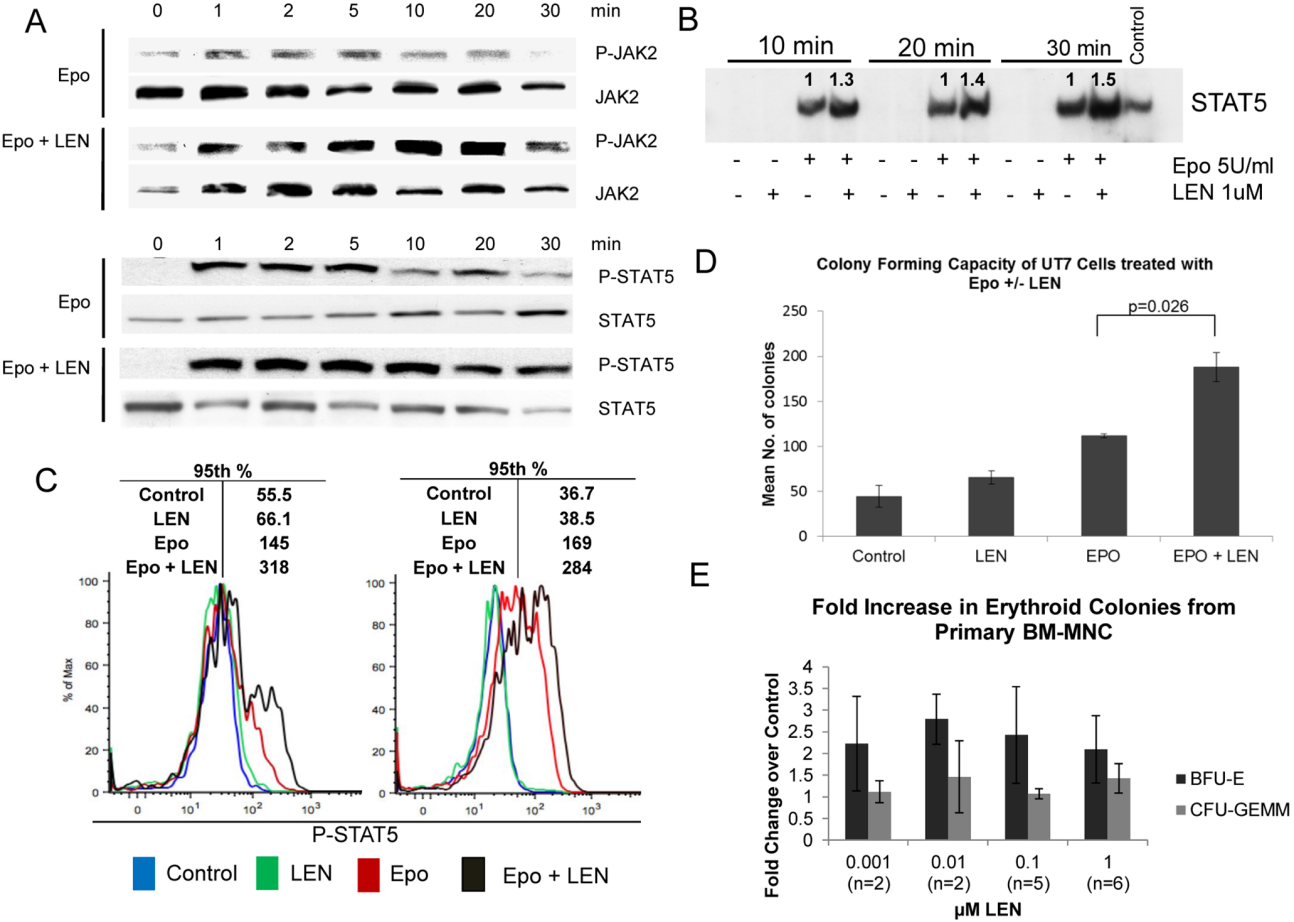
Lenalidomide enhances EpoR signaling and erythroid colony growth. (A) Western blot showing increased and prolonged JAK2 and STAT5 phosphorylation in UT7 cells treated with 1 U/ml rhEpo and 1 µM lenalidomide pretreatment for 1 h. (B) STAT5 EMSA showing increased and sustained binding of STAT5 to DNA in UT7 cells pretreated with lenalidomide. K562 cells were used as controls. Numbers represent densitometry analysis of lenalidomide + rhu-Epo treatment compared to Epo treatment alone at each time point. (C) Flow cytometry histograms showing an increase in P-STAT5 in primary erythroid progenitors identified as CD45^dim^, CD71^high^, and GlyA^low^ with MFI of the 95^th^% provided. (D) Colony forming capacity of UT7 cells pretreated with or without lenalidomide. 5 U/ml of rhEpo was added to the indicated wells with each performed in triplicate. Colonies were counted after 7 d incubation and are represented as mean ± SE. (E) Colony forming capacity of MDS BM-MNC showing more than a 2-fold increase of BFU-E after lenalidomide pretreatment without a marked increase in mixed lineage CFU-GEMM.

To determine if lenalidomide enhances EpoR signaling, we treated UT7 cells with rhEpo alone and after treatment with 1 µM lenalidomide. JAK2 phosphorylation following rhEpo stimulation was enhanced and prolonged following lenalidomide pretreatment (Figure 4A). Similar findings were observed with STAT5 phosphorylation in which lenalidomide pretreatment both augmented and extended the duration of phosphorylation of the transcription factor (Figure 4A). We next evaluated whether lenalidomide increased binding of STAT5 to DNA using electrophoretic mobility shift assay (EMSA) to influence transcriptional response. We found that lenalidomide increased and sustained the binding of the transcription factor to DNA in UT7 cells when given prior to rhEpo (Figure 4B). These data indicate lenalidomide potentiates EpoR signaling in UT7 erythroid progenitors through increased STAT5 DNA binding, which may account for the promotion of erythroid gene specific transcriptional response described by Ebert et al [2]. We next investigated the effects of lenalidomide on EpoR signaling following rhEpo stimulation in primary MDS erythroid progenitors by flow cytometry. Primitive erythroid precursors characterized by CD45^dim^, CD71^high^ and GlyA^low^, were analyzed for changes in STAT5 phosphorylation. Lenalidomide pretreatment markedly increased STAT5 phosphorylation in response to rhEpo stimulation in MDS erythroid progenitors compared to rhEpo treatment alone. Figure 4C illustrates flow diagrams from two MDS patients showing a marked increase in P-STAT5 mean florescence intensity (MFI) with the combined treatment. Of the 12 non-del(5q) MDS patients analyzed who had no previous exposure to lenalidomide, we found an increase in the 95th percentile P-STAT5 MFI in 7 of the 12 with lenalidomide pretreatment compared to rhEpo treatment alone. We utilized the 95th percentile read out due to the small population of cells that shift, which is more biologically representative than MFI in the absence of a uniform population shift [16]. Of the 7 patients with increased P-STAT5, the number of cells positive for P-STAT5 ranged from 0.585% to 66.5% with rhEpo treatment only, and increased an average of 79.1%±8.25% with lenalidomide pretreatment. To determine if potentiation of EpoR signaling by lenalidomide influenced colony-forming capacity, we assessed colony recovery with and without lenalidomide treatment in UT7 cells and primary MDS bone marrow mononuclear cells. UT7 cells were pretreated for 2 h with lenalidomide, then plated in cytokine containing methylcellulose with rhEpo. Combination treatment with lenalidomide and rhEpo yielded a significant increase in colony number compared to rhEpo alone (p = 0.026) (Figure 4D). To further verify this, we performed colony formation assays using primary MDS BM-MNCs, with 2 to 6 patients evaluated at each indicated concentration of lenalidomide. We found more than a two-fold increase in erythroid burst forming units (BFU-E) following lenalidomide pretreatment, whereas there was minimal change in the number of mixed colony forming units-granulocyte, erythrocyte, monocyte, and megakaryocyte (CFU-GEMM) or (CFU-GM), suggesting an erythroid specific effect (Figure 4E). Although we did not find a dose dependent increase in erythroid colonies, these data are consistent with previous findings by Ebert et al. where maximal augmentation of BFU-E recovery with lenalidomide was observed at concentrations ≤1 µM [17].

### ROCK inhibition prevents F-actin polymerization and raft assembly induced by lenalidomide

**Figure 5:**
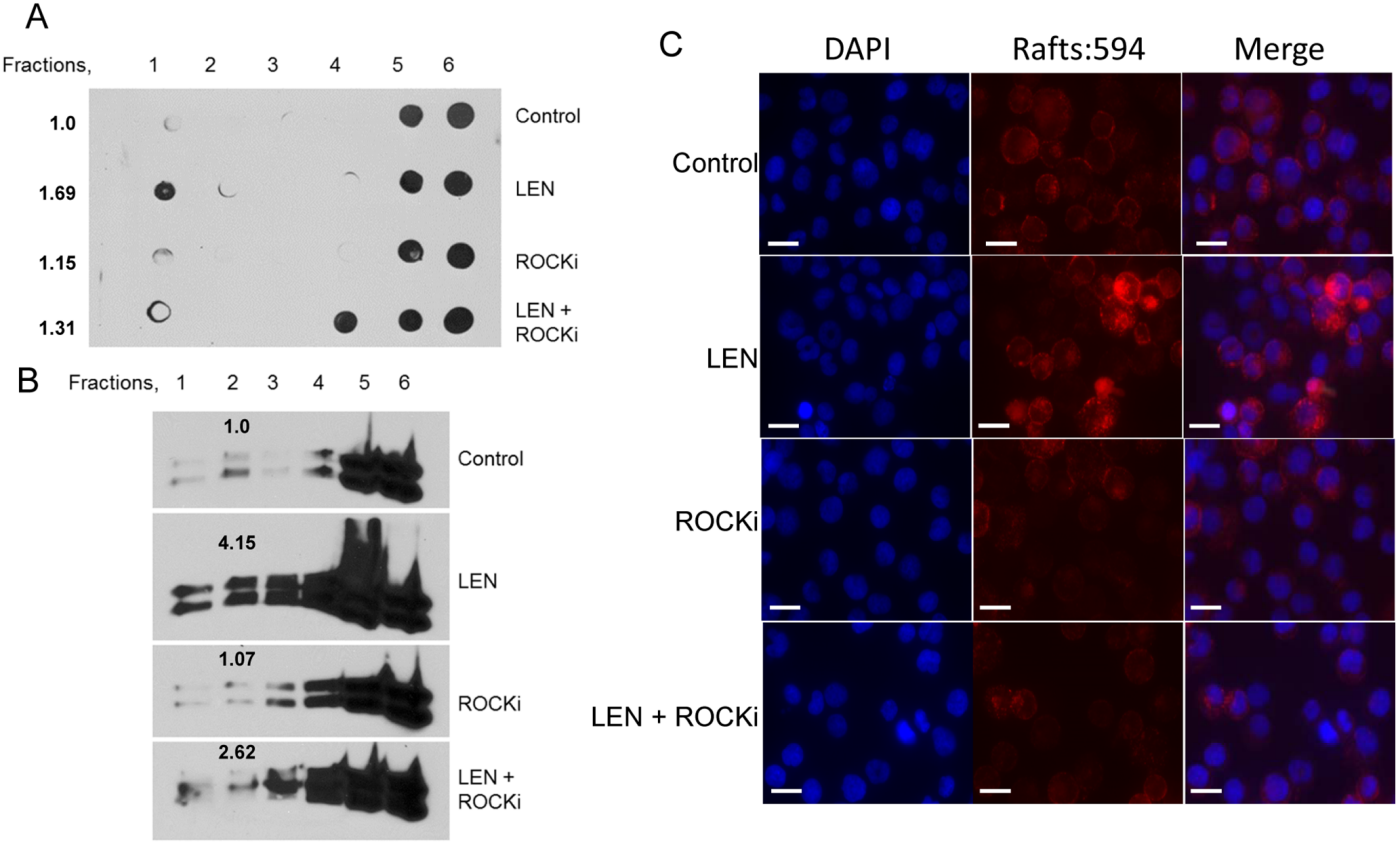
ROCK inhibition abrogates lenalidomide induced accumulation of lipid rafts. (A) Dot blot detection of GM-1 in UT7 cells treated with lenalidomide with or without the ROCK inhibitor, Y-27632 (ROCKi), pretreatment showing a decrease in raft fractionation with combination treatment. Rafts are located in fractions 1 and 2, while non-raft fractions are 4–6. Numbers represent densitometry analysis of combined raft fractions 1 and 2. (B) Western blot of Lyn kinase indicative of raft fractionation (fractions 1 and 2) showing an increase after lenalidomide treatment that is partially blocked with ROCKi pretreatment. Numbers represent densitometry analysis of combined raft fractions 1–3. (C) Immunofluorescence of rafts (red), DAPI (blue), and merged image showing inhibition of lenalidomide induced raft formation with ROCKi pretreatment. Bars represent 20 µm.

**Figure 6:**
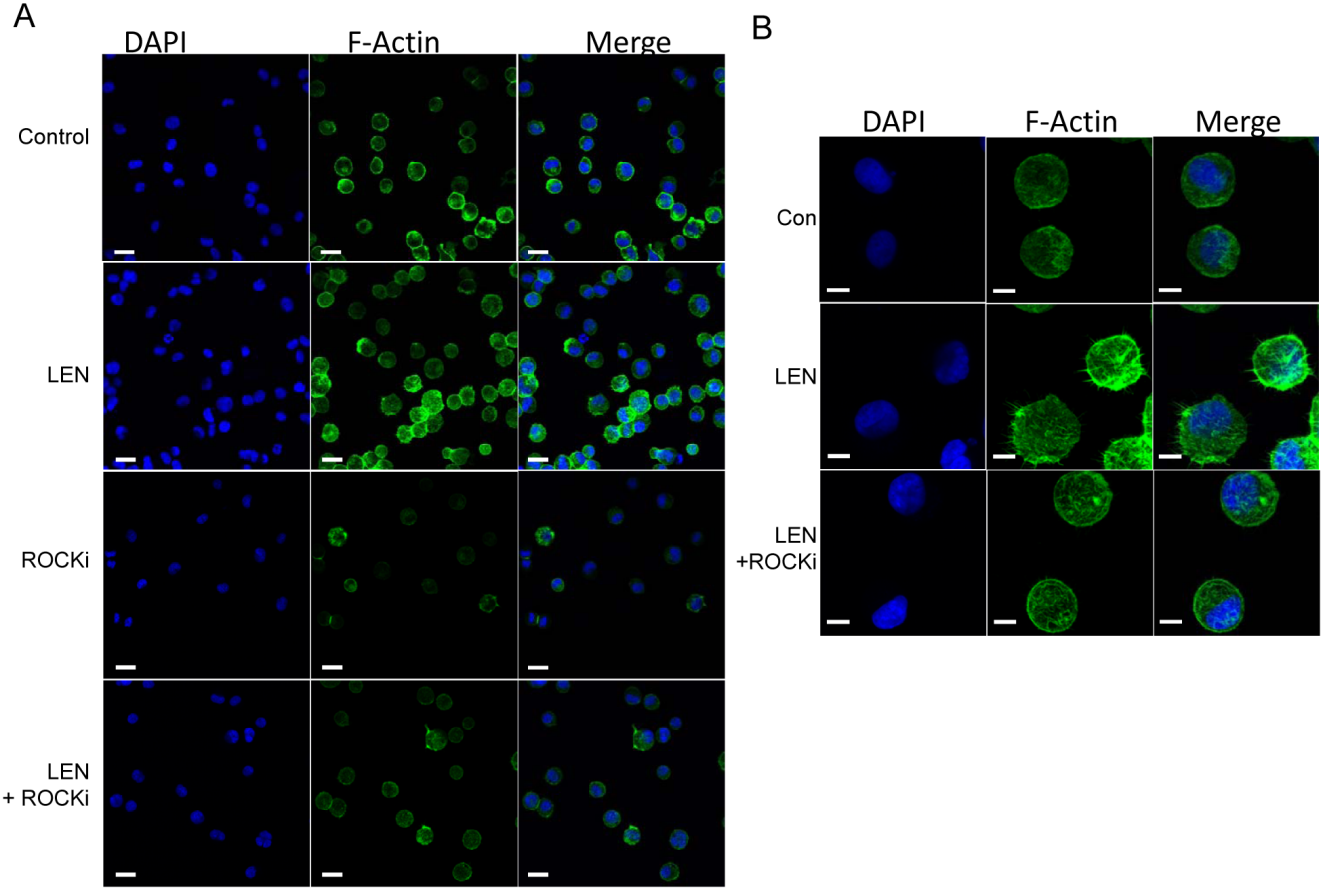
Lenalidomide induces actin polymerization that is blocked by ROCK inhibition. (A) Confocal microscopy at 630x and (B) 630x with 4x zoom of phalloidin (green) used to detect actin polymerization, DAPI (blue), and merged image. Lenalidomide treatment induced actin polymerization that was inhibited by pre-treatment with ROCKi, Y-27632. Bars represent 20 µm (A) and 10 µm (B).

We previously reported that rhEpo induction of lipid raft assembly was Rac GTPase dependent. Inhibition of the Rho-associated protein kinase, ROCK, and Rac GTPase inhibited recruitment of EpoR into the raft fractions after Epo stimulation [4]. To determine whether ROCK was similarly involved in the induction of lipid rafts by lenalidomide, UT7 cells were treated with lenalidomide either with or without pretreatment with 100 µM of the ROCK inhibitor, Y-27632, for 30 min. Pretreatment with Y-27632 inhibited the induction of lipid rafts (fractions 1–3) by lenalidomide as shown by GM-1 dot blot analysis (Figure 5A) and by western blot of Lyn kinase from isolated cellular fractions (Figure 5B). These findings were confirmed by confocal microscopy imaging of lipid rafts (Figure 5C). Moreover, lenalidomide induction of actin filament polymerization was also inhibited by the ROCK inhibitor, as analyzed by confocal microscopy of phalloidin stained cells (Figures 6A–B). These findings implicate ROCK in the induction of actin filament polymerization and membrane lipid raft assembly by lenalidomide.

## Discussion

The data shown here provide new insight into abnormalities in the EpoR signaling platform that underlie impaired Epo responsiveness in MDS erythroid precursors. EpoR signal fidelity is dependent upon receptor residence within membrane raft microdomains, whereas disruption of lipid rafts impairs signal capacity. Here we show that MDS erythroid precursors display deficient lipid raft formation, and importantly, that lenalidomide promotes lipid raft assembly and raft aggregate formation to enhance erythroid colony forming capacity in response to Epo stimulation. Lenalidomide-induced raft formation was associated with recruitment of the EpoR into raft microdomains accompanied by the incorporation of the key signaling effectors JAK2, STAT5, and Lyn kinase. Of particular importance, the transmembrane tyrosine phosphatase, CD45, which acts to extinguish receptor signaling by dephosphorylating JAK2 and the EpoR, was re-partitioned to non-raft fractions. Such dynamic changes in the raft compartment should enhance EpoR signal fidelity by optimizing the signaling platform. Indeed, lenalidomide pretreatment enhanced both JAK2 and STAT5 phosphorylation following Epo stimulation in UT7 cells as well as primary MDS erythroid precursors, and similarly increased and sustained STAT5 DNA binding. These findings may explain observations by Ebert et. al. [2] that bone marrow mononuclear cells from lenalidomide-responsive MDS patients underexpress a set of erythroid differentiation genes whose expression was restored following lenalidomide exposure. Our investigations indicate that lenalidomide promotes lipid raft assembly and improves EpoR signal fidelity to enhance receptor signaling and transcription factor DNA binding. These data also provide a biological rationale for the results of a recent Groupe Francais des Myelodysplasies (GFM) study group phase II trial that compared treatment with lenalidomide to combined treatment with lenalidomide and epoetin beta in lower risk, transfusion dependent MDS patients who had failed prior treatment with recombinant erythropoietins [18]. Results of this study showed that the combination yielded a significantly higher rate of erythroid response compared to lenalidomide alone (40% vs. 24.3%; p = 0.04), indicating that the addition of lenalidomide restored Epo responsiveness in a significant proportion of patients. Flow cytometry detection of erythroid subpopulations in which EpoR/STAT5 signaling was augmented by lenalidomide therefore might serve as a potential biomarker for response. Our preliminary studies show that lipid raft aggregates were significantly larger in lenalidomide responders, suggesting that capacity for raft assembly may influence lenalidomide response. This, as well as the relationship between *ex* *vivo* induction of raft assembly and lenalidomide response, is currently under investigation in an ongoing Eastern Cooperative Oncology Group sponsored phase III intergroup trial [E2905].

Lipid raft assembly is driven in part by actin filament polymerization. Our investigations show that induction of raft assembly by lenalidomide is dependent upon F-actin polymerization, in a Rho-associated protein kinase (ROCK)-dependent manner. Precisely how lenalidomide activates ROCK remains unclear and merits further investigation [8], [9], [19]. Abnormalities in actin polymerization have been implicated in MDS pathogenesis, and may underlie the observed deficiency in raft assembly. Recent investigations have shown that expression of the unconventional Rac activating guanine nucleotide exchange factor (GEF), DOCK4, is decreased in MDS patients compared to age-matched controls [20]. DOCK4 is a member of the CDM (*C. elegans* Ced-5, mammalian DOCK180 and *D. melanogaster* myoblast city) family of proteins, which are regulators of adherens junctions and cell migration. The *DOCK4* promoter is hypermethylated in MDS, with consequent silencing of gene expression [20]. A recent report showed that *DOCK4* silencing in MDS was associated with diminished F-actin polymerization [21]. Moreover, decreased *DOCK4* expression was associated with increased erythrocyte fragility which was confirmed by gene knockdown experiments in primary erythroid progenitors [21]. These findings provide a plausible pathobiological rationale for the ineffective erythropoiesis in MDS in which intrinsic cytoskeletal abnormalities arising from decreased DOCK4 initiated polymerization of actin impairs lipid raft assembly and growth factor receptor signaling. Rac GTPase dependent raft assembly, which in our investigations is partially rescued by lenalidomide, further supports this notion. The effects of lenalidomide on DOCK4 expression and activity warrant further investigation.

Although lenalidomide has been previously reported to activate GTPases, the mechanism by which this occurs is unknown [9]. Recent findings have shown that IMiDs bind to the cereblon E3 ubiquitin ligase complex to inhibit ligase function, which accounts in part for the teratogenicity of thalidomide, as well as the anti-proliferative effects in multiple myeloma [22], [23]. However, more recent reports suggest that lenalidomide instead binds to CRBN to induce its E3 ligase activity, causing degradation of the Ikaros transcription factors and accounting for the anti-tumor activity in myeloma [24]–[26]. These conflicting data suggest that cereblon may be ambiguous in its function with respect to not only cellular pathway, but also to cell lineage. The role of cereblon, if any, in lenalidomide-induced raft formation remains unclear. Transfection approaches to alter cereblon gene expression also disrupt raft integrity (data not shown), thus perhaps only the creation of a stable knockdown could properly address the role of cereblon in lenalidomide induction of raft assembly.

In addition to modifying the E3 ligase activity of cereblon, we recently reported that lenalidomide inhibits the activity of the human homolog of the murine double minute-2 E3 ubiquitin ligase, MDM2 [27]. Inhibition of MDM2 auto-ubiquitination stabilizes the protein, permitting binding to and degradation of p53 in del(5q) clones [27]. These findings suggest that lenalidomide may have broader E3 ligase inhibitory effects. It is possible that lenalidomide may activate GTPases through inhibition of E3 ligases involved in their degradation. Several ligases are known to ubiquitinate RhoA, including SMAD ubiquitination regulatory factor 1, SMURF1 and the CRL3 complex (Cullin-RING ubiquitin ligase). [28], [29] Additionally, the HECT ligase, HACE1, has recently been implicated in the ubiquitination and degradation of Rac1 [30]. Investigation of the effects of lenalidomide on these E3 ligases and consequent effects on GTPase activation and actin cytoskeletal reorganization is currently underway.

## Methods

### Reagents and cells

UT7 cells, acquired from American Type Culture Collection (ATCC, Manassas, VA), were maintained in alpha-MEM supplemented with 20% FBS, 1% penicillin/streptomycin solution, and 5 ng/ml GM-CSF in a humidified incubator with 5% CO_2_. Bone marrow mononuclear cells (BM-MNC) were isolated from 11 MDS patients with written consent on University of South Florida Institutional Review Board approved protocol (IRB #106744) using Ficoll-Paque Plus (GE Healthcare, Little Chalfont, UK) method and from 3 normal donors purchased from Lonza Walkersville Inc. (Walkersville, MD). Lenalidomide was purchased through Fisher Scientific (Pittsburgh, PA). CT-B:HRP was purchased from Sigma-Aldrich (St. Louis, MO) Recombinant human erythropoietin (rhEpo) was purchased from Stemcell Technologies (Vancouver, BC). JAK2, STAT5, cKit, and Lyn antibodies were purchased from Santa Cruz Biotechnology (Santa Cruz, CA). CD71 antibody was purchased from Abcam (Cambridge, MA). CD45 antibody was purchased from BD Biosciences (San Jose, CA). EpoR antibody was provided by Amgen (Thousand Oaks, CA). Secondary antibodies were purchased from Life Technologies Corporation (Carlsbad, CA). ProLong Anti-fade reagent with DAPI was purchased from Life Technologies. ROCK inhibitor, Y-27632 dihydrochloride monohydrate, was purchased from Sigma-Aldrich. Alexa-Fluor 488 phalloidin was purchased from Life Technologies (Carlsbad, CA).

### Lipid raft isolation

Lipid rafts were isolated as previously described [4]. Briefly, cells were lysed in 0.75% Triton X-100 in TNE buffer [25 mM Tris pH7, 150 mM EDTA, 1 mM DTT, 150 mM NaCl, and 1 Complete EDTA-free protease inhibitor tablet from Roche (Indianapolis, IN) per 20 mL buffer]. Cells were then passed through a 27 G needle and left on ice for 5 min. Lysates were pipetted below a decreasing concentration gradient of Optiprep purchased from Sigma-Aldrich. Samples were ultracentrifuged at 20000 rpm for 20 h in a Beckman Coulter (Fullerton, CA) Optima L-90 K ultracentrifuge. Fractions were pipetted off one by one and used for either western blotting and/or dot blotting.

### Western blotting

Fractions isolated after raft isolation or whole cell lysates were resolved by SDS-PAGE then transferred to PVDF membranes. The membranes were blocked for 30 min in 5% dry milk solution in PBST (PBS with 0.1% Tween20) and incubated with the indicated antibodies. Membranes were developed using ECL or ECL+ according to manufacturer’s protocol (GE Healthcare, Little Chalfont, UK). Densitometry analysis was performed using AlphaEaseFC software (Genetic Technologies, Inc. Miami, FL).

### Dot blotting

Dot blotting was performed as previously described [4]. Briefly, 5 µL of each fraction isolated from ultracentrifugation was pipetted onto a nitrocellulose membrane. The membrane was then washed in PBS and blocked for 30 min in 0.3% PBS-Tween20 solution. Membranes were incubated with CT-B:HRP overnight then washed three times in PBS with 0.3% PBS Tween20. Membranes were developed using ECL according to manufacturer’s protocol. The specificity of CT-B for lipid rafts was confirmed in our previous publication using methyl-β-cyclodextrin (MβCD) to disrupt rafts [4].

### Colony formation assay

BM-MNC were pretreated with the indicated concentrations of lenalidomide for 24 h prior to plating in methylcellulose containing cytokine complete media with Epo at a cell density of 500,000 cells per/mL. Plates were incubated for 14 d at 37°C with 5% CO_2,_ in a humidified incubator. Colonies were differentiated by morphology and counted. UT7 cells were either untreated, or pretreated for 2 h with 1 µM lenalidomide, then washed and plated at 2000 cells per well in methylcellulose with 10% FBS at a final volume of 200 µL. For Epo conditions, 5 U/mL was added to the methylcellulose. Cells were incubated for 7d at 37°C, 5% CO_2,_ in a humidified incubator and colonies were scored.

### Electrophoretic mobility shift assay

Electrophoretic mobility shift assay (EMSA) was performed as previously described [31]. Briefly, UT7 cells were pretreated with 1 µM lenalidomide for 1 h prior to the addition of 5 U/ml rhEpo. Nuclear extracts collected at the indicated times were incubated with the 32-P labeled MGFe oligonucleotide probes that bind STAT5, and STAT5 DNA binding assessed by EMSA.

### Flow cytometry

BM-MNC were isolated from patients who had written consent on IRB approved research protocols using Ficoll-Paque. The cells were treated with EPO with or without lenalidomide (1 h pretreatment at 1 µM) and fixed with BD Cytofix (BD Biosciences). Cells were stored at −80°C until staining was performed. Cells were washed with PBS then permeabilized in pre-warmed BD Phosflow Perm Buffer III (BD Biosciences) for 30 min on ice in the dark. Cells were washed and stained with CD71:APC, GlyA:FITC, CD45:PerCP-Cy5.5, and P-STAT5:PE antibodies (BD Bioscience) for 30 min in the dark. Cells were washed and resuspended in BD staining buffer for flow analysis. The samples were run on a FACScalibur flow cytometer.

### Immunofluorescence

Raft immunofluorescence was performed as previously described [4]. Briefly, treated cells were stained with Vibrant Lipid Raft Labeling kit (Life Technologies Corporation) per manufacturer’s protocol, then cytospun at 450 rpm for 5 min. Slides were then fixed with BD Cytofix (BD Biosciences) for 10 min at 37°C. Slides were washed in PBS and a drop of ProLong Anti-fade reagent with DAPI was added with a cover slip. Micrographs were taken using an upright Zeiss Axio-ImagerZ.1 microscope with a 63x/1.40NA oil immersion objective, and DAPI, FITC, and Rhodamine filter cubes with an AxioCam MRm CCD camera using Axiovision version 4.6 software suite (Carl Zeiss, Inc. Thornwood, NY) or with a Leica TCS SP5 AOBS laser scanning confocal microscope through a 20X/0.5NA or 63X/1.40NA Plan Apochromatic oil immersion objective lens (Leica Microsystems CMS GmbH, Germany). For confocal microscopy, 405 Diode, Argon 488, and HeNe 543, 594, or 633 laser lines were applied to excite the samples and tunable emissions were used to minimize crosstalk between fluorochromes. Image sections at 0.5 µm were captured with photomultiplier detectors and maximum projections were prepared with the LAS AF software version 2.1.0 (Leica Microsystems CMS GmbH, Germany).

For F-actin staining, cells were treated, then fixed and cytospun. Slides were permeabilized in 0.5% Triton-X for 5 min at room temperature then washed and blocked in 2% BSA-PBS. Cells were then stained with Alexa Fluor 488 phalloidin according to manufacturer’s protocol (Life Technologies Corporation). Cells were washed, then ProLong Anti-fade reagent with DAPI was added with a coverslip, and micrographs were taken on the confocal microscope. For primary cell immunofluorescence experiments, cells were treated and rafts were stained as described above. Before adding DAPI, cells were blocked and stained with CD71 (1∶200) and cKit (1∶20) antibodies for 1 h at room temperature. Slides were then washed and incubated in Alexa Fluor 488 or Alex Fluor 647 secondary antibodies (1∶1000) for 1 h at room temperature. Micrographs were taken by confocal microscopy. All parameters were kept the same between patients and conditions were kept consistent using InSpeck. Microscope Image Intensity Calibration beads (Life Technologies Corporation).

### Statistical analysis

Lipid Raft Analysis was performed with Definiens Developer version 2.0 (Definiens AG, Munich, Germany). First, CD71 positive cells were identified and segmented using an automatic threshold algorithm on the Alexa 488 channel layer. The segmentation was further refined by using a size threshold to remove background debris. Next the cKit+ cells were identified from CD71 segmented cells. Nuclei identified individual cells using automatic threshold algorithm of the DAPI channel. Finally, an intensity threshold of 50 grayscale values was used on the lipid raft staining channel to identify and segment lipid raft aggregates. The following features were extracted from the above segmentation: number of nuclei, area of nuclei, number of lipid raft aggregates, area of lipid raft aggregates, and intensity of lipid raft aggregates. Cells were pooled for each condition and patient and presented as mean ± SE was reported. P-values were determined using unpaired student t-tests.
